# Clinical Outcomes of a New-Generation Surgical Aortic Bioprosthesis: A Noninferiority Study

**DOI:** 10.1016/j.xjon.2025.101571

**Published:** 2025-12-23

**Authors:** Daniela Geisler, Zsuzsanna Arnold, Rudolf Seemann, Martin Grabenwöger, Markus Mach

**Affiliations:** a Department of Cardiac and Vascular Surgery, Clinic Floridsdorf, Vienna, Austria; b Karl Landsteiner Institute of Cardiovascular Research, Vienna, Austria; c Institute of Head and Neck Diseases, Evangelic Hospital, Vienna, Austria; d Sigmund Freud Private University, Medical Faculty, Vienna, Austria; e University Clinic of Cardiac and Thoracic Aortic Surgery, Medical University Vienna, Vienna, Austria; f Department of Cardiac Surgery, University Hospital Graz, Graz, Austria

**Keywords:** bioprosthetic heart valve, Inspiris Resilia, Magna Ease, SAVR, structural valve deterioration

## Abstract

**Objectives:**

The Inspiris Resilia valve features novel anticalcification technology and based on the Magna Ease design (Edwards Lifesciences) has shown promising preclinical durability. However, its clinical benefit remains uncertain. This study presents the first noninferiority analysis comparing Inspiris Resilia and Magna Ease valves in a real-world cohort. Our aim is to evaluate clinical outcomes of the Inspiris Resilia compared with the Magna Ease valve, regarding noninferiority in all-cause mortality at up to 7 years follow-up.

**Methods:**

A retrospective noninferiority study was conducted at a single center in Vienna, including 1704 consecutive patients who underwent surgical aortic valve replacement between October 2008 and January 2024. After propensity score matching for age, sex, European System for Cardiac Operative Risk Evaluation II, valve size, isolated versus combined surgery, and periprocedural mortality, 554 patients were included in each group.

**Results:**

In the Cox proportional hazard model, the Inspiris Resilia valve demonstrated noninferiority in overall survival (hazard ratio, 0.791; 95% CI, 0.546-1.146). Cumulative survival at 1, 3, 5, and 7 years was 94.6%, 93.1%, 91.1%, and 86.8% for the Inspiris Resilia, and 94.8%, 91.9%, 87.7%, and 84.2% for the Magna Ease, respectively. Both groups displayed low rates of bioprosthetic valve failure. Mean age was 67.0 ± 8.6 years (Inspiris Resilia) and 67.7 ± 8.9 years (Magna Ease), with comparable European System for Cardiac Operative Risk Evaluation II values (3.2 ± 3.4 vs 3.3 ± 3.2).

**Conclusions:**

This study establishes the Inspiris Resilia valve as at least equivalent to the Magna Ease in mid-term outcomes, while offering the potential to guide future surgical valve selection.

**Figure:**
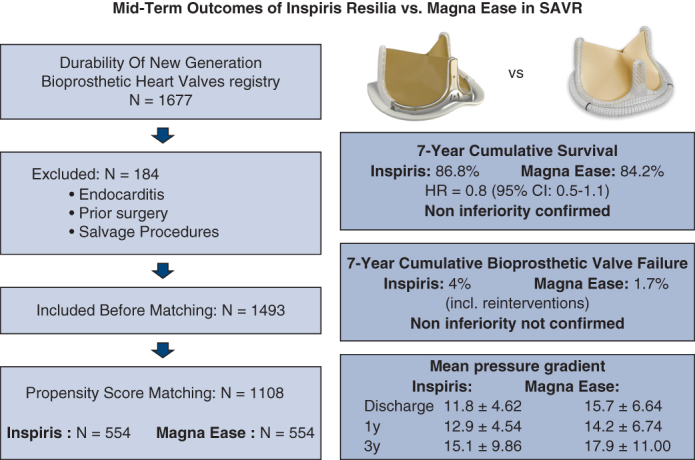
IR shows noninferior survival, better hemodynamics, and promising mid-term durability.

**Central Message**

In a 7-year analysis, the IR valve showed noninferior survival and favorable hemodynamics versus the ME, supporting its use in surgical valve selection and long-term durability.

**Perspective**

This study shows noninferiority of the IR valve compared with the ME valve in mid-term survival, supporting its use as a next-generation surgical bioprosthesis. Although early durability is promising, long-term outcomes remain to be confirmed. Extended follow-up is needed to assess whether anticalcification strategies reduce SVD and extend valve lifespan.

The first xenograft heart valve constructed from porcine pericardium was implanted in the aortic position by Carpentier in 1965. At that time, early failure was primarily driven by immunogenic responses; however, Carpentier and colleagues^1^ recognized the importance of tissue preservation techniques in improving graft durability. Since then, advances in tissue engineering, preservation strategies, and valve design have significantly enhanced the longevity and performance of bioprosthetic heart valves (BHVs).^2^^,^^3^

Structural valve deterioration (SVD) remains the leading cause of BHV failure.^4^ This issue has gained increasing relevance as BHVs are now being implanted in younger patients, largely due to their advantages over mechanical valves—namely, the absence of a lifelong need for anticoagulation, favorable hemodynamic profiles, and a lower risk of thromboembolic complications.^5^, ^6^, ^7^ Additionally, the expanding availability of interventional options has steadily increased the use of BHVs.^4^ Ongoing innovation in valve technologies is focused on extending durability, with the goal of enabling true lifetime management strategies.^8^ The Perimount Magna Ease (ME; Edwards Lifesciences) is a third-generation bovine pericardial BHV in the Carpentier-Edwards series, and it has demonstrated excellent clinical outcomes worldwide.^9^^,^^10^ In patients aged less than 65 years, the actuarial rate of calcific degeneration has been reported at up to 9.7% at 10-year follow-up, with a survival of 92.6%.^9^, ^10^, ^11^ In 2017, the Inspiris Resilia (IR) valve, featuring novel anticalcification “Resilia” tissue, was introduced to the European market as the ME's successor.^12^^,^^13^

Four cohort studies have recently compared the IR and ME valves.^14^, ^15^, ^16^, ^17^ Two focused on early (30-day) mortality as a short-term outcome. Shala and Niclauss^14^ reported equal mortality (1/59 vs 1/66), whereas another study showed 0.5% mortality in the IR group (n = 605) compared with 2% in the ME group (n = 296).^15^ Bernard and colleagues^17^ found survival of 94% (IR) versus 91% (ME) at 30 months and identified 2 cases of moderate SVD in the IR group at 2-year follow-up. In contrast, Francica and colleagues^16^ observed no cases of SVD in the IR group and a 2.3% rate in the ME group over 3 years, with no mortality reported in either cohort.

Given the limited number of comparative studies to date, demonstrating noninferiority is an essential step before long-term superiority claims can be made. To our knowledge, this is the first study to assess the noninferiority of the IR valve relative to the ME valve over a mid-term follow-up period. Accordingly, we aim to evaluate and compare clinical outcomes between these 2 BHVs over a follow-up of up to 7 years.

## Material and Methods

### Study Design

A retrospective cohort study was designed to prove the noninferiority of the IR valve compared with the ME valve. Patient data were obtained from the “Durability Of New Generation Bioprosthetic Heart Valves” registry (EK 22-260-VK), which includes all Edwards Lifesciences valve implantations performed at the Department of Cardiac and Vascular Surgery—initially located at Hietzing Hospital and, after relocation in 2019, at Clinic Floridsdorf, Vienna. The registry was initiated and maintained by the first author. The study protocol was approved by the local ethics committee (Reference Number EK 24-204-VK, 24/12/2024). Written informed consent was not required, in accordance with national legislation and institutional policy. Although the first author received a personal research grant, the company had no access to the data and did not influence the study design, analysis, or reporting in any way.

### Study Cohort

Between October 2008 and April 2019, 929 consecutive patients (317 female, 612 male) underwent isolated or combined surgical aortic valve replacement (SAVR) using the ME valve. From July 2017 to January 2024, 748 patients (224 female, 524 male) received the IR valve. All procedures followed institutional protocols aligned with European Society of Cardiology/European Association for Cardio-Thoracic Surgery guidelines for the management of valvular heart disease.^18^

Patients were excluded if they had endocarditis within 3 months before surgery, a valve size more than 27 mm (because 29 mm was not used in the ME group), or previous aortic or cardiac surgery, or underwent emergency/salvage procedures (eg, aortic dissection). After applying these criteria, 847 patients in the ME group and 646 patients in the IR group were eligible.

Mortality data were collected from electronic health records, obituary notices, and telephone follow-up. Echocardiographic data were retrieved from institutional records or the referring cardiology centers.

### Propensity Score Matching

**Figure 1:**
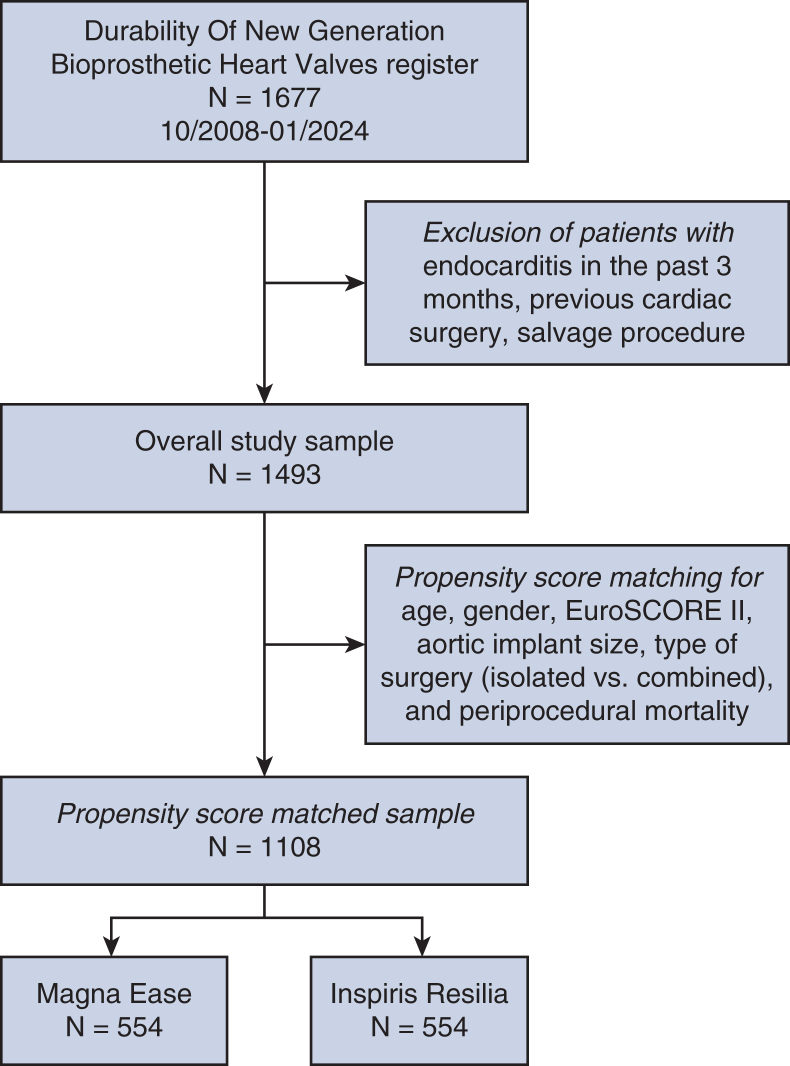
Patient inclusion flowchart diagram illustrating the study population from initial registry (N = 1677) through exclusions and final PS-matched sample (n = 1108), split into IR (n = 554) and ME (n = 554) groups.

To adjust for baseline differences, propensity score (PS) matching was performed using age, sex, European System for Cardiac Operative Risk Evaluation (EuroSCORE) II, aortic valve size, and surgery type (isolated vs combined). Although periprocedural mortality did not differ in the initial database (Table 1), the first matching step revealed an imbalance reflecting historical differences in perioperative care. Periprocedural mortality was defined according to Valve Academic Research Consortium 3 (VARC-3) criteria as death occurring within 30 days post procedure or during index hospitalization. Therefore, surgery period (pre- vs post relocation) was incorporated as an additional covariate to account for improvements in perioperative care and infrastructure rather than mortality itself. This refinement reduced historical bias while maintaining the validity of the matching process. After 1:1 nearest-neighbor matching, 554 patient pairs (n = 1108) were included (Figure 1).

**Table 1:** Matching parameters before and after propensity score matching

Parameter	Before matching			After matching		
	IR	ME	Test	IR	ME	Test
N=	646	847		554	554	
Age	66.5 ± 8.8	68.5 ± 9.5	t = −3.866 df = 1435.8 *P =* .0001	67.0 ± 8.6	67.7 ± 8.9	t = −1.3621 df = 1103.4 *P =* .173
Gender			chi-square = 2.5629 df = 1 *P =* .1094			chi-square = 0.425 df = 1 *P =* .514
male	451 (69.8%)	557 (65.8%)		390 (70.4%)	379 (68.4%)	
Female	195 (30.2%)	290 (34.2%)		164 (29.6%)	175 (31.6%)	
EuroSCORE II, %	3.3 ± 4.4	2.4 ± 2.9	t = 4.6466 df = 1066 *P* < .001	3.2 ± 3.4	3.3 ± 3.2	t = −0.63852 df = 1098.7 *P =* .523
Aortic implant size			chi-square = 5.8464 df = 4 *P =* .2109			chi-square = 1.9086 df = 4 *P =* .753
19	33 (5.1%)	57 (6.7%)		27 (4.9%)	36 (6.5%)	
21	149 (21.7%)	215 (25.4%)		122 (22.0%)	128 (23.1%)	
23	222 (34.4%)	272 (32.1%)		185 (33.4%)	177 (31.9%)	
25	176 (27.2%)	203 (24.0%)		153 (27.6%)	144 (26.0%)	
27	75 (11.6%)	100 (11.8%)		67 (12.1%)	69 (12.5%)	
Type of surgery			chi-square = 0.15277 df = 1 *P =* .6959			chi-square = 0.01455 df = 1 *P =* .904
Isolated	290 (44.9%)	390 (46.0%)		254 (45.8%)	251 (45.3%)	
Combined	356 (55.1%)	457 (54.0%)		300 (54.2%)	303 (54.7%)	
Periprocedural mortality	17 (2.6%)	37 (4.4%)	chi-square = 2.6925 df = 1 *P =* .1008	17 (3.1%)	15 (2.7%)	chi-square = 0.03217 df = 1 *P =* .857

Summary of the variables used for PS matching in the IR and ME groups, including age, sex, EuroSCORE II, valve size, surgery type, and periprocedural mortality. Matching success is demonstrated by comparable distributions postmatching. Group comparisons were performed using Welch's 2-sample *t* test for continuous variables and Pearson's chi-square test with Yates' continuity correction for categorical variables. *IR,* Inspiris Resilia; *ME,* Magna Ease; *df,* degrees of freedom; *EuroSCORE,* European System for Cardiac Operative Risk Evaluation.

### End Points

The primary end point was all-cause mortality at 1, 3, 5, and 7 years postimplantation, reported as cumulative survival. The secondary end point was bioprosthetic valve failure (BVF), defined as irreversible stage 3 hemodynamic deterioration (mean pressure gradient >40 mmHg, severe aortic regurgitation) or valve reintervention due to SVD, according to VARC-3. The main hypothesis was noninferiority of IR relative to ME in terms of all-cause mortality.

Hemodynamic performance was assessed using Doppler echocardiography. The following parameters were evaluated: left ventricular ejection fraction, mean and peak transvalvular gradients, peak velocity, and prosthetic valve regurgitation. Assessments were made postoperatively and at follow-up intervals. Severe patient–prosthesis mismatch (PPM) was defined as an indexed effective orifice area (iEOA) less than 0.65 cm^2^/m^2^ and moderate PPM as an iEOA between 0.65 and 0.85 cm^2^/m^2^ in patients with a body mass index below 30 kg/m^2^. In patients with a body mass index 30 kg/m^2^ or greater, severe PPM was defined as an iEOA 0.55 cm^2^/m^2^ or greater and moderate PPM as an iEOA between 0.56 and 0.70 cm^2^/m^2^.^19^^,^^20^

### Sample Size Calculation

The sample size was determined a priori based on the noninferiority hypothesis for the primary end point of all-cause mortality. Assuming a true hazard ratio (HR) of 0.9, a noninferiority margin of HR 1.25 or less, a 1-sided alpha level of 0.05%, and 95% power, the Schoenfeld method indicated that approximately 100 events (corresponding to ∼1003 patients in total, assuming a 10% event rate over 5 years) would be required to demonstrate noninferiority. Given the available cohort of 1108 matched patients, the study was adequately powered to test the primary end point. However, secondary end points such as BVF were expected to be rarer events and therefore underpowered for formal noninferiority testing.

### Statistical Analysis

All statistical analyses were conducted using R (version 4.3.2).^21^ PS matching was performed using the MatchIt package (version 4.5.5) with 1:1 nearest-neighbor matching, a caliper width of 0.1, and no replacement.^22^ Cox proportional hazards models were used to assess both primary and secondary end points. A noninferiority margin of 1.25 for the HR was applied according to prior literature. This threshold reflects that a 25% or less relative difference in mortality is considered clinically acceptable when balanced against potential procedural or hemodynamic advantages. There were no missing data for survival analyses, whereas hemodynamic outcomes were analyzed based on the available cases only. Cumulative event rates were estimated by the Kaplan–Meier method and compared with the log-rank test.

## Results

### Overall Population and the Propensity Score–matched Sample

After applying exclusion criteria, the overall population included 1493 patients. Patients receiving the IR valve were younger (66.5 ± 8.8 vs 68.5 ± 9.5 years, *P* = .0001) and had a higher risk profile (EuroSCORE II: 3.3 ± 4.4 vs 2.4 ± 2.9, *P* < .0001) compared with those receiving the ME valve.

The final study cohort included 1108 patients (554 patients per group) after PS matching. All matched parameters—age, sex, EuroSCORE II, aortic implant size, and procedure type (isolated vs combined surgery)—were well balanced, with no statistically significant differences (Table 1).

Additional unmatched baseline characteristics of the PS-matched cohort are presented in Table 2. Patients in the IR group had a higher prevalence of diabetes mellitus, whereas the ME group had a higher prevalence of smoking and associated chronic obstructive pulmonary disease. The ME group also had significantly higher preoperative creatinine levels and a greater prevalence of cerebrovascular and coronary artery disease.

**Table 2:** Unmatched baseline characteristics in the propensity score–matched population

Parameter	IR N = 554	ME N = 554	*P* value
	n (%)/Mean ± SD	n (%)/Mean ± SD	
Weight, kg	83 ± 16.6	83.7 ± 16.3	.485
Height, cm	172 ± 9.1	172 ± 9.5	.509
BMI, kg cm^−2^	28.9 ± 4.9	28.2 ± 4.8	.221
Diabetes mellitus	185 (33.4%)	148 (26.7%)	.018
Arterial hypertension	397 (71.7%)	422 (76.2%)	.101
Dyslipidemia	316 (57%)	340 (61.4%)	.160
Active/former smoker	145 (26.2%)	177 (31.9%)	.040
COPD	70 (12.6%)	107 (19.3%)	.003
Creatine level preoperative (mg/dL)	0.954 ± 0.58	1.07 ± 0.53	.001
Dialysis preoperative	2 (0.4%)	3 (0.5%)	1.000
Previous pacemaker	12 (2.2%)	11 (2%)	1.000
Peripheral vascular disease	37 (6.7%)	51 (9.2%)	.149
Cerebrovascular disease	36 (6.5%)	69 (12.5%)	.001
Coronary artery disease	223 (40.3%)	260 (46.9%)	.029
Prior MCI <90 d	26 (4.7%)	20 (3.6%)	.451
NYHA			.353
I	46 (8.3%)	45 (8.1%)	
II	215 (38.8%)	213 (38.4%)	
III	287 (51.8%)	282 (50.9%)	
IV	6 (1.1%)	14 (2.5%)	
Severe AV stenosis	468 (84.5%)	464 (83.8%)	.805
Mean gradient, mm Hg	41.6 ± 23.3	37.7 ± 27.7	.010
Aortic regurgitation			.017
Severe	33 (6%)	20 (3.6%)	
Moderate	62 (11.2%)	63 (11.4%)	
Mild	106 (19.1%)	77 (13.9%)	
Trivial	155 (28%)	153 (27.6%)	
None	198 (35.7%)	241 (43.5%)	
Mitral regurgitation			<.001
Severe	11 (2%)	7 (1.3%)	
Moderate	16 (2.9%)	26 (4.7%)	
Mild	64 (11.6%)	83 (15%)	
Trivial	322 (58.1%)	219 (39.5%)	
None	141 (25.5%)	219 (39.5%)	
Tricuspid regurgitation			<.001
Severe	6 (1.1%)	6 (1.1%)	
Moderate	5 (0.9%)	10 (1.8%)	
Mild	29 (5.2%)	25 (4.5%)	
Trivial	303 (54.7%)	171 (30.9%)	
None	211 (38.1%)	342 (61.7%)	

Baseline clinical variables not included in the matching algorithm. Statistically significant differences were found in diabetes, smoking, chronic obstructive pulmonary disease, creatinine levels, and cerebrovascular and coronary artery disease. *IR,* Inspiris Resilia; *ME,* Magna Ease; *COPD,* chronic obstructive pulmonary disease; *MCI,* myocardial infarction; *NYHA,* New York Heart Association; *AV,* aortic valve.

Intraoperative variables are reported in Table 3. Crossclamp times were significantly longer in the ME group, which also had a higher frequency of concomitant coronary artery bypass grafting. Conversely, the IR group underwent more root and ascending aortic replacements. Concomitant procedures are detailed in Table 3.

**Table 3:** Intraoperative parameters

Parameter	IR N = 554	ME N = 554	*P* value
	n (%)/Mean ± SD	n (%)/Mean ± SD	
CPB time, min	120 ± 84.50	125 ± 43.20	.159
Crossclamp time, min	81.1 ± 42.70	85.7 ± 29.10	.035
Concomitant procedures			
CABG	160 (28.9%)	197 (35.6%)	.021
Root replacement	45 (8.1%)	23 (4.2%)	<.001
Ascending aorta replacement	76 (13.7%)	18 (3.2%)	<.001
Aortic annular enlargement	32 (5.8%)	23 (4.2%)	.269
Mitral valve repair	12 (2.2%)	18 (3.2%)	.503
Mitral valve replacement	18 (3.2%)	22 (4%)	.421
Tricuspid reconstruction	10 (1.8%)	14 (2.5%)	.536
AF correction surgery	27 (4.9%)	40 (7.2%)	.130
Carotid endarterectomy	9 (1.6%)	9 (1.6%)	1.000
IABP	5 (0.9%)	10 (1.8%)	.298
ECMO	9 (1.6%)	7 (1.3%)	.801

Operative variables including crossclamp time, bypass time, and the frequency of concomitant procedures such as coronary artery bypass grafting, aortic root/ascending aorta replacement, and valve repairs. *IR,* Inspiris Resilia; *ME,* Magna Ease; *CPB,* cardiopulmonary bypass; *CABG,* coronary artery bypass grafting; *AF,* atrial fibrillation; *IABP,* intra-aortic balloon pump; *ECMO,* extracorporeal membrane oxygenation.

### Clinical Outcomes

#### Primary end point – Overall survival

**Figure 2:**
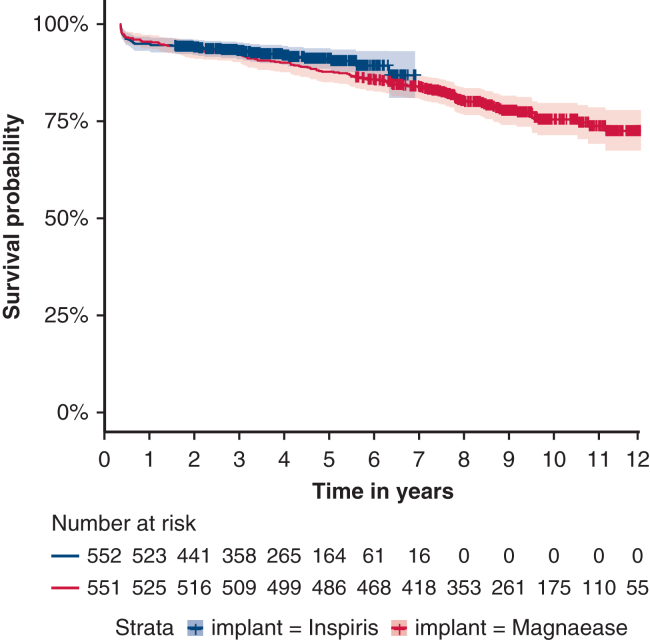
Kaplan–Meier curve for overall survival. Comparison of cumulative all-cause mortality in the IR and ME groups using a Cox proportional hazards model. At 7 years, the IR valve demonstrated noninferiority with cumulative survival of 86.8% compared with 84.2% for the ME valve (HR, 0.791; 95% CI, 0.546-1.146; *P = .*21). The individual graph *lines* were truncated before the number of subjects at risk decreased to fewer than 10.

In the Cox proportional hazards model, the IR valve demonstrated noninferiority with respect to overall survival (HR, 0.791; 95% CI, 0.546-1.146; Figure 2). Cumulative survivals at 1, 3, 5, and 7 years were 94.6% (number at risk [NAR] = 524), 93.1% (NAR = 359), 91.1% (NAR = 165), and 86.8% (95% CI, 81.0%-93.0%, NAR = 17) in the IR group, respectively, and 94.8% (NAR = 526), 91.9% (NAR = 510), 87.7% (NAR = 487), and 84.2% (95% CI, 81.2%-87.3%, NAR = 419) in the ME group, respectively.

Because the ME cohort included longer follow-up, the 10-year and 12-year cumulative survivals were 77.3% (95% CI, 73.6-81.2, NAR = 176) and 72.5% (95% CI, 67.5-77.9, NAR = 56), respectively. Mean follow-up duration was 3.8 ± 1.8 years for the IR group and 8.4 ± 3.1 years for the ME group.

#### Secondary end point – Bioprosthetic valve failure

Noninferiority for BVF was not demonstrated HR 2.390 (95% CI 0.684–8.350). Cumulative BVF rates in the IR group were 0.4% (NAR = 522), 1.3% (NAR = 355), 4.0% (NAR = 162), and 4.0% (NAR = 17) at 1, 3, 5, and 7 years, respectively. In the ME group, rates were 0.2% (NAR = 524) at 1 year, 0.4% (NAR = 508) at 3 years, 1.2% (NAR = 484) at 5 years, and 1.7% (NAR = 413) at 7 years. Extended follow-up in the ME cohort showed 10-year and 12-year cumulative BVF rates of 3.3% and 6.7%, respectively. During the follow-up period, reinterventions were performed in 11 patients (2.0%) in the IR group and 15 patients (2.7%) in the ME group (Table 4).

**Table 4:** Follow-up hemodynamics and reintervention rates

Parameter	N=	IR N = 554	N=	ME N = 554	*P* value
		n (%)/Mean ± SD		n (%)/Mean ± SD	
Discharge aortic mean gradient, mm Hg	474	11.8 ± 4.62	388	15.7 ± 6.64	<.001
Discharge aortic peak gradient, mm Hg	498	20.6 ± 7.92	390	28.8 ± 27.40	<.001
Discharge Vmax, m/s	492	2.24 ± 0.39	388	2.56 ± 0.49	<.001
PPM no	554	427 (77%)	554	408 (73%)	.392
Moderate		122 (22%)		139 (25%)	
Severe		5 (0.1%)		7 (0.1%)	
1 y aortic mean gradient, mm Hg	72	12.9 ± 4.54	37	14.2 ± 6.74	.288
1 y aortic peak gradient, mm Hg	114	16.4 ± 12.80	39	23.6 ± 10.80	<.001
1 y Vmax, m/s	115	1.69 ± 1.15	43	2.39 ± 0.56	<.001
3 y aortic mean gradient, mm Hg	78	8.18 ± 9.17	47	16.5 ± 10.40	<.001
3 y aortic peak gradient, mm Hg	83	15.2 ± 16.60	56	27.8 ± 19.70	<.001
3 y Vmax, m/s	84	1.41 ± 1.35	57	2.48 ± 0.80	<.001
5 y aortic mean gradient, mm Hg	40	15.1 ± 9.86	49	17.9 ± 11.00	.21
5 y aortic peak gradient, mm Hg	40	27.1 ± 16.80	53	29.3 ± 17.90	.561
5 y Vmax, m/s	41	2.45 ± 0.76	55	2.59 ± 0.72	.384
Reintervention		11 (2%)		15 (2.7%)	.199
Redo SAVR		9 (1.6%)		5 (0.9%)	
ViV TAVR		2 (0.4%)		10 (1.8%)	
Reasons for reintervention					.058
Aortic regurgitation	11	1 (9.1%)	15	0 (0%)	
Endocarditis	11	4 (36.4%)	15	3 (20%)	
PVL	11	2 (18.2%)	15	0 (0%)	
Subvalvular stenosis	11	1 (9.1%)	15	0 (0%)	
SVD	11	3 (27.3%)	15	12 (80%)	

Echocardiographic performance at discharge and follow-up intervals. Reintervention events and cumulative bioprosthetic valve failure rates are also reported. *IR,* Inspiris Resilia; *ME,* Magna Ease; *PPM,* patient–prosthesis mismatch; *SAVR,* surgical aortic valve replacement; *ViV,* valve-in-valve; *TAVR,* transcatheter aortic valve replacement; *PVL,* paravalvular leak; *SVD,* structural valve deterioration.

#### Postoperative parameters

Postoperative outcomes were comparable between the matched groups, with low overall rates of major complications (Table 5). A significantly higher incidence of new-onset postoperative atrial fibrillation (both paroxysmal and permanent) was observed in the IR group.

**Table 5:** Postoperative outcomes

Parameter	IR N = 544	ME N = 544	*P* value
	n (%)/Mean ± SD	n (%)/Mean ± SD	
ICU stay, d	4.7 ± 11.7	4.0 ± 12.2	.286
Surgical revision for bleeding/tamponade	21 (3.8%)	20 (3.6%)	1.000
Neurologic event (type 1 stroke)	3 (0.5%)	2 (0.4%)	1.000
TIA (type 3 stroke)	11 (2%)	5 (0.9%)	.208
Dialysis (newly required)	14 (2.5%)	14 (2.5%)	1.000
New pacemaker implantation	20 (3.6%)	21 (3.8%)	1.000
New-onset atrial fibrillation	110 (19.9%)	53 (9.6%)	<.001
Sternal wound infection requiring VAC therapy	3 (0.5%)	4 (0.7%)	1.000
Readmit ≤30 d from date of procedure	12 (2.2%)	9 (1.6%)	.660

Clinical outcomes during the postoperative period, including intensive care unit stay, new-onset atrial fibrillation, stroke, dialysis, pacemaker implantation, and surgical complications. Notably, atrial fibrillation was significantly more frequent in the IR group. *IR,* Inspiris Resilia; *ME,* Magna Ease; *ICU,* Intensive care unit; *TIA,* transient ischemic attack; *VAC,* vacuum-assisted closure.

#### Hemodynamic parameters

Echocardiographic hemodynamic criteria favored the IR valve, which demonstrated lower transvalvular gradients and better flow characteristics at discharge and throughout 3-year follow-up (Table 4). Moderate or severe PPM was apparent in 22.1% in the IR group (n = 127) and 25.1% in the ME group (n = 146; Table 4).

## Discussion

To our knowledge, this is the first study to demonstrate the noninferiority of the IR valve in terms of overall survival when compared with its predecessor, the ME, while also providing insight into mid-term structural valve performance. Although the ME has well-established long-term data, the long-term superiority of the IR remains to be proven. Because clinically relevant rates of BVF often occur after 10 to 20 years, the current follow-up duration was insufficient to confirm noninferiority for BVF.^2^

Although the literature commonly uses the term “SVD” to describe irreversible stage 3 valve dysfunction, it is critical to focus on clinically meaningful end points. For this reason, we adopted the VARC-3 definition of BVF, which includes bioprosthetic valve dysfunction, irreversible hemodynamic valve deterioration, and aortic valve reintervention, thus emphasizing outcome relevance over pure morphology.^20^

In the current landscape of lifetime management strategies, selecting an optimal aortic valve prosthesis involves balancing long-term durability against reintervention risk and overall survival. A growing trend toward bioprosthetic valves with extended durability reflects this evolving paradigm.^23^ Earlier generations of Carpentier-Edwards Perimount pericardial valves have shown excellent durability. Notably, Bourguignon and colleagues^24^ and Johnston and colleagues^2^ reported outcomes up to 20 years post implantation. However, in those studies, only 14.4% of patients were alive at 20 years (mean age: 70.7 ± 10.4 years), and Johnston and colleagues estimated a 76% probability of death before explant in patients with a mean age of 71 years. In patients younger than 60 years, explant rates reached 46% by 20 years—highlighting the limitations in durability and the importance of further valve advancements.^2^

Since its market release in 2008, the ME valve has been increasingly used in younger patients, improving long-term survival. In a series by Piperata and colleagues,^10^ 12-year survival was 81% in patients aged less than 65 years and 45% in those aged 65 years or more, with a 12-year SVD rate of 7% and 10% reoperation rate among 2148 patients.^10^ In our ME cohort, 10- and 12-year BVF rates were 3.3% and 6.7%, respectively—lower than those reported by Piperata and colleagues. Likewise, Francica and colleagues^9^ found 84.3% survival at 10 years in patients aged less than 65 years and 68.2% in an older cohort. The ME valve continues to show favorable outcomes also when compared with other pericardial BHVs.^25^^,^^26^

The IR valve represents a technological evolution, offering a new tissue preservation strategy designed to reduce calcification.^12^ The COMMENCE trial—currently the longest follow-up study on IR—reported 89.4% survival at 5 years and 85.4% at up to 7 years (mean age: 66.9 ± 11.6 years), closely aligning with our observed survival of 91.1% at 5 years and 86.8% at 7 years. The reported SVD rate, defined as dysfunction or deterioration of the operated valve excluding infection, was 0.7% at 7 years.^27^^,^^28^ Likewise, Bartus and colleagues^13^^,^^29^ observed 83.4% 5-year survival (mean age: 65.3 ± 13.5 years) with no SVD events. Although these findings underscore IR's mid-term durability, few studies have directly compared IR with earlier Perimount generations or other contemporary BHVs.^14^, ^15^, ^16^, ^17^

In our analysis, cumulative BVF incidence over 7 years remained low in both groups—4% in IR and 1.7% in ME. Noninferiority could not be statistically confirmed for BVF, but given the low absolute number of BVF events in both cohorts, the study was not statistically powered to demonstrate noninferiority for this secondary end point.^10^^,^^27^ Notably, the distribution of reintervention causes differed between groups, with 36.4% of reinterventions in the IR cohort attributed to endocarditis—potentially reflecting contemporary clinical challenges rather than valve-related failure (Table 5).^30^ In contrast, SVD accounted for only 27.3% (3 cases) of reinterventions in the IR cohort compared to 80% (12 cases) in the ME cohort, suggesting a difference in failure mechanisms rather than an increased overall failure rate. Overall, reintervention rates were low and not significantly different, mirroring results from the COMMENCE trial, which reported 12 reinterventions—including 2 for SVD—over 7 years.^27^

The role of PPM on long-term performance has been extensively discussed in the literature. A large meta-analysis identified PPM as a significant risk factor for SVD and reduced long-term survival in 122,989 patients.^31^ Although moderate PPM is frequently observed, as seen in our sample with 22% in the IR and 25% in the ME group, its clinical impact appears to be minimal, because the absolute differences in clinical outcomes are small.^32^ However, in the context of lifetime management strategies, PPM has regained attention, because subsequent reinterventions affect the effective orifice area and thus influence the degree of PPM.

In this context, direct comparisons between the IR and ME valves are essential to assess the clinical value of IR's anticalcification technology, especially considering the unchanged valve design.^15^, ^16^, ^17^^,^^33^ The ongoing INSPIRIS RESILIA Durability Registry (INDURE) focuses on patients aged less than 60 years (mean age: 53.5 years), and early results have shown excellent hemodynamic performance, with a mean aortic valve gradient of 12.6 mmHg—closely matching our findings of 11.8 mmHg at discharge and 12.9 mmHg at 1 year.^34^ Notably, despite identical design, the IR consistently demonstrated improved hemodynamic parameters compared with the ME, in line with prior reports.^15^^,^^17^

### Limitations

Despite its strengths, this study has several limitations. The primary limitation is the lack of a standardized and comprehensive longitudinal echocardiographic follow-up to assess SVD over time. This is partly due to the fact that most follow-up echocardiography was performed in private outpatient settings, where data collection may be inconsistent or incomplete. Additionally, follow-up routines were disrupted during the COVID-19 pandemic, potentially introducing information bias due to missing or unevenly gathered data. Nevertheless, loss to follow-up remains a common challenge in cardiac surgery trials, as previously reported by Beaver and colleagues,^27^ who found that only one-third of patients were available for follow-up at 7 years.

Second, all patients in the ME group underwent surgery before the clinic's relocation to a new facility equipped with an intermediate care unit. As a result, periprocedural mortality in this group may have been influenced by differences in perioperative care related to the time of recruitment, rather than by the prosthesis itself. This potential historical bias was addressed through inclusion of recruitment period in the PS matching.

Third, additional historical bias may have been introduced by evolving patient characteristics over time. Compared with the IR group, the ME cohort had a higher prevalence of smoking, chronic obstructive pulmonary disease, and coronary artery disease, which likely influenced the types of concomitant procedures performed. As a single-center retrospective study, the findings are subject to limitations in generalizability and potential selection bias, despite rigorous matching and robust data validation methods.

## Conclusions

This is the first study to demonstrate noninferiority of the IR valve compared with its predecessor, the ME valve, in terms of all-cause mortality at mid-term follow-up. Our large, single-center cohort offers valuable real-world insights into the clinical performance of the IR valve over a 7-year period.

Nonetheless, important questions remain, particularly regarding the long-term incidence of BVF, extended survival, and the evolving contribution of SVD in next-generation bioprostheses. Further prospective studies with long-term follow-up are needed to address these uncertainties and guide future surgical valve selection.

### Declaration of Generative Artificial Intelligence and Artificial Intelligence–Assisted Technologies in the Writing Process

During the preparation of this work, the author used ChatGPT (OpenAI) to assist with language refinement and formatting suggestions. After using this tool, the authors reviewed and edited the content as needed and take full responsibility for the content of the publication.

## Conflict of Interest Statement

Dr Geisler received a personal research grant from Edwards Lifesciences. All other authors reported no conflicts of interest.

The *Journal* policy requires editors and reviewers to disclose conflicts of interest and to decline handling or reviewing manuscripts for which they may have a conflict of interest. The editors and reviewers of this article have no conflicts of interest.
